# Hyper inflammatory syndrome following COVID-19 mRNA vaccine in children: A national post-authorization pharmacovigilance study

**DOI:** 10.1016/j.lanepe.2022.100393

**Published:** 2022-04-29

**Authors:** Naïm Ouldali, Haleh Bagheri, Francesco Salvo, Denise Antona, Antoine Pariente, Claire Leblanc, Martine Tebacher, Joëlle Micallef, Corinne Levy, Robert Cohen, Etienne Javouhey, Brigitte Bader-Meunier, Caroline Ovaert, Sylvain Renolleau, Veronique Hentgen, Isabelle Kone-Paut, Nina Deschamps, Loïc De Pontual, Xavier Iriart, Christelle Gras-Le Guen, François Angoulvant, Alexandre Belot, “French Covid-19 Paediatric Inflammation Consortium”£ and the “French Pharmacovigilance network”*

**Affiliations:** a Assistance Publique-Hôpitaux de Paris, Department of general paediatrics, paediatric infectious disease and internal medicine, Robert Debré university hospital, Université de Paris, Paris, France; b Infectious Diseases Division, CHU Sainte Justine - Montreal University, Montreal, Quebec, Canada; c ACTIV, Association Clinique et Thérapeutique Infantile du Val-de-Marne, Créteil, France; d Université de Paris, INSERM UMR 1123, ECEVE, Paris, France; e Department of Medical and Clinical Pharmacology, Regional Pharmacovigilance Center, CIC 1436, Toulouse University Hospital, France; f INSERM, BPH, U1219, Team Pharmacoepidemiology, Université de Bordeaux, 33000 Bordeaux, France; g Pole de Santé Publique, Service de Pharmacologie Médicale, Regional pharmacovigilance center of Bordeaux, CHU de Bordeaux, 33000 Bordeaux, France; h Santé Publique France, Agence nationale de Santé publique, Saint-Maurice, France; i Team Pharmacoepidemiology, U1219 BPH Research Center, Bordeaux University, INSERM, Bordeaux, France; j Regional pharmacovigilance center of Strasbourg, HUS, Strasbourg, France; k Marseille University hospital, Clinical pharmacology department Regional Pharmacovigilance Center of Marseille, France; l Aix-Marseille University, INSERM UMR 1106, Marseille, France; m Centre Hospitalier Intercommunal, Research Center, Université Paris Est, IMRB-GRC GEMINI, Créteil, France; n Hospices Civils de Lyon, Paediatric Intensive Care Unit, Hopital Femme, Mère Enfant, University of Lyon, Bron, France; o EA 7426 "Pathophysiology of Injury-Induced Immunosuppression", University Claude Bernard Lyon 1, Hospices Civils of Lyon, Lyon, France; p Department of Paediatric Hematology-Immunology and Rheumatology, Necker-Enfants Malades Hospital, AP-HP, Paris, France, Reference center for Rheumatic, AutoImmune and Systemic diseases in children (RAISE), Paris, France; q Laboratory of Immunogenetics of Paediatric Autoimmunity, Imagine Institute, Paris University, Inserm U 1163, Paris, France; r Department of Pediatric Cardiology, Hôpital Timone Enfant, AP-HM, Marseille, France; s Aix-Marseille University, MMG, INSERM, Marseille, France; t Pediatric Intensive Care Unit, Necker Hospital, AP-HP, Paris University, Paris, France; u Paris University, Paris EA7323, France; v General Pediatrics department, Versailles Hospital, Paris, France; w CEREMAIA (French reference center for auto-inflammatory diseases and inflammatory amyloidosis), Paris, France; x Pediatric Rheumatology Department, Bicêtre Hospital, APHP, University of Paris Saclay, Kremlin Bicêtre, France; y General Pediatrics department, Saint-Malo Hospital, Saint-Malo, France; z General Pediatrics and Pediatric emergency department, Jean Verdier Hospital, Bondy, France; aa Department of Pediatric and Adult Congenital Cardiology, Bordeaux University Hospital, Pessac, France; ab Institut Hospitalo-Universitaire Liryc, Electrophysiology and Heart Modeling Institute, Fondation Bordeaux Université, Bordeaux, Pessac, France; ac INSERM, Centre de recherche Cardio-Thoracique de Bordeaux, Bordeaux, France; ad Department of Pediatric Emergency Care, Nantes University Hospital, Nantes F-44000, France; ae Obstetrical, Perinatal and Pediatric Epidemiology Research team, Center of Research in Epidemiology and Statistics, Université de Paris, INSERM, Paris F-75004, France; af Inserm CIC 1413, Nantes University Hospital, Nantes F-44000, France; ag INSERM, Centre de Recherche des Cordeliers, UMRS 1138, Sorbonne Université, Université de Paris, Paris, France; ah Hospices Civils de Lyon, Paediatric Nephrology, Rheumatology, Dermatology, Hopital Femme, Mère Enfant and Centre International de Recherche en Infectiologie / INSERM U1111, Bron, France; ai National Reference Center for Rheumatic and AutoImmune and Systemic Diseases in Children (RAISE), Lyon, France; aj International Center of Research in Infectiology, Lyon University, INSERM U1111, CNRS UMR 5308, ENS, UCBL, 69007 Lyon, France

**Keywords:** COVID-19 mRNA vaccine, BNT162b2, Multisystem inflammatory syndrome in children, Hyper-inflammatory syndrome, child, SARS-COV-2

## Abstract

**Background:**

Multisystem inflammatory syndrome in children (MIS-C) is the most severe clinical entity associated with pediatric SARS-CoV-2 infection with a putative role of the spike protein into the immune system activation. Whether COVID-19 mRNA vaccine can induce this complication in children is unknown. We aimed to assess the risk of hyper-inflammatory syndrome following COVID-19 mRNA vaccine in children.

**Methods:**

We conducted a post-authorization national population-based surveillance using the French enhanced pharmacovigilance surveillance system for COVID-19 vaccines. All cases of suspected hyper-inflammatory syndrome following COVID-19 mRNA vaccine in 12–17-year-old children between June 15^th^, 2021 and January 1^st^, 2022, were reported. Cases were reviewed according to WHO criteria for MIS-C. The reporting rate of this syndrome was compared to the MIS-C rate per 1,000,000 12–17-year-old children infected by SARS-CoV-2.

**Findings:**

Up to January 2022, 8,113,058 COVID-19 mRNA vaccine doses were administered to 4,079,234 12–17-year-old children. Among them, 12 presented a hyper-inflammatory syndrome with multisystemic involvement. Main clinical features included male predominance (10/12, 83%), cardiac involvement (10/12, 83%), digestive symptoms (10/12, 83%), coagulopathy (7/12, 58%), cytolytic hepatitis (6/12, 50%), and shock (5/12, 42%). 4/12 (33%) required intensive care unit transfer, and 3/12 (25%) hemodynamic support. All cases recovered. In eight cases, no evidence of previous SARS-CoV-2 infection was found. The reporting rate was 1.5 (95%CI [0.8; 2.6]) per 1,000,000 doses injected, i.e. 2.9 (95%CI [1.5; 5.1]) per 1,000,000 12–17-year-old vaccinated children. As a comparison, 113 MIS-C (95%CI [95; 135]) occurred per 1,000,000 12–17-year-old children infected by SARS-CoV-2.

**Interpretation:**

Very few cases of hyper-inflammatory syndrome with multi-organ involvement occurred following COVID-19 mRNA vaccine in 12–17-year-old children. The low reporting rate of this syndrome, compared to the rate of post-SARS-CoV-2 MIS-C in the same age-group, largely supports the vaccination in a context of an important circulation of SARS-CoV-2.

**Funding:**

ESPID Fellowship Award; Grandir–Fonds de Solidarité Pour L'enfance.

**Research in context**

**Evidence before this study**

We searched PubMed for articles published in English up to February 15, 2022, using the terms “Multisystem inflammatory syndrome” or “Paediatric inflammatory multisystem syndrome” or “MIS-C” or “PIMS” and “COVID-19 mRNA vaccine”. Multisystem inflammatory syndrome in children (MIS-C) is the most severe clinical entity associated with SARS-CoV-2 infection in children. The pathophysiology of this syndrome remains unclear, and concerns have been raised regarding the possibility that exposure to Spike protein antigen with mRNA vaccines could induce a multisystemic hyper inflammatory response. Several case reports of MIS-C following mRNA vaccines involving children of adults have been reported, and a recent surveillance investigation from USA reported several cases of MIS-C in children who received a COVID-19 vaccine. Population-based pharmacovigilance studies are required to assess this risk and compare it to the rate of post SARS-CoV-2 MIS-C.

**Added value of this study**

Using a French national post-authorization pharmacovigilance study, we identified 12 children aged 12 to 17 years old who developed a hyper inflammatory syndrome following COVID-19 mRNA vaccine, with a multisystemic involvement. In 8 cases, no evidence of previous SARS-CoV-2 infection was found. 10 out of 12 cases displayed a cardiac involvement . We observed a male predominance (10/12, 83% of cases), as reported for post-vaccinal myocarditis. The reporting rate was 1.5 (95%CI [0.8; 2.6]) per 1,000,000 doses injected, i.e. 2.9 (95% CI [1.5; 5.1]) per 1,000,000 12–17-year-old vaccinated children. As a comparison, 113 MIS-C (95%CI [95; 135]) occurred per 1,000,000 12–17-year-old children infected by SARS-CoV-2 in the same population. Clinical features (hepatic involvement, inflammatory parameters, cytopenia) slightly differed from post-SARS-CoV-2 MIS-C, along with short-term outcomes (less PICU transfer than MIS-C).

**Implications of all the available evidence**

This study shows that very few cases of hyper-inflammatory syndrome, with multisystemic involvement, may be observed after COVID-19 mRNA vaccine in children. The incidence of this event, compared to the rate of MIS-C following SARS-CoV-2 infection in the same age-group population, may help policy makers estimating the benefit-risk balance of COVID-19 mRNA vaccine in children. Large pharmacovigilance studies from other countries are required to precisely determine the role of COVID-19 mRNA vaccine in this syndrome, and to delineate the clinical spectrum of this entity compared to post-SARS-CoV-2 MIS-C.

Alt-text: Unlabelled box

## Introduction

Multisystem inflammatory syndrome in children (MIS-C) is a novel clinical entity first described in April 2020.^1^, ^2^, ^3^, ^4^, ^5^ Its association with SARS-CoV-2 infection has been documented, with a previous infection occurring 4 to 6 weeks before MIS-C onset.^4^, ^5^, ^6^, ^7^ The main clinical features of MIS-C are frequent acute cardiac dysfunction, shock, multi organ failure that often require pediatric intensive care unit transfer and hemodynamic support.^8^ Thus, numerous studies showed that MIS-C is by far the most severe clinical syndrome associated with SARS-CoV-2 infection in children and a leading source of morbidity related to SARS-CoV-2 in this age group.^8^^,^^9^

The pathophysiology of this disease remains unknown, but previous investigations showed that MIS-C is characterized by a cytokine storm^10^ associated with a superantigen-like activation of T cells with an expansion of Vβ21.3-expressing T cells which is not seen in toxic shock syndrome, Kawasaki disease or other COVID-19 features.^11^, ^12^, ^13^, ^14^ Notably, SARS-CoV2 spike harbors a motif located in the receptor binding domain that is predicted *in silico* to interact with Vβ region in T cells. Whether antigenic exposure limited to the Spike protein can lead to similar dysregulated immune response remains unknown.

Several COVID-19 mRNA vaccines have been shown to be efficacious and well tolerated in adults, and have been introduced since December 2020.^15^ Post-authorization studies confirmed their major impact on SARS-CoV-2 epidemics,^16^ with very few serious adverse events reported to date.^17^^,^^18^ Israel reported a risk of post-mRNA vaccine myocarditis of 1.76 per 100,000 individual, mainly affecting young males.^19^ In children, the immunogenicity, efficacy, as well as frequency of adverse events have been assessed in trials involving thousands of 12-17-year-old children.^20^ Based on these studies, the Food and Drug Administration (FDA) and European Medicines Agency (EMA) authorized formulations of mRNA COVID-19 vaccine for ages 12-17-year. However, rare serious adverse events following immunization could not be detected by these clinical trials, which were not designed to assess the risk of rare hyper-inflammatory complications. Especially, whether exposure to SARS-CoV-2 antigens due to mRNA vaccine can induce MIS-C is unknown.

Given the lower burden of SARS-CoV-2 related diseases in children compared to adults, elucidating the safety profile of mRNA vaccine, especially regarding MIS-C, is of critical interest to establish its benefit-risk balance in this population. In this context, monitoring post-vaccine MIS-C has been identified as a priority by the FDA, the EMA, and the French National Agency for Medicines and Health Products safety (ANSM).^21^, ^22^, ^23^ Several cases reports of children with MIS-C following mRNA vaccine recently raised important concerns regarding this potential vaccine-related adverse event.^24^, ^25^, ^26^, ^27^

Using a well-established national pharmacovigilance surveillance system coordinated by ANSM,^28^^,^^29^ we aimed to evaluate the potential association of COVID-19 mRNA vaccine and subsequent hyper-inflammatory syndrome in children.

## Methods

### Study design and settings

We conducted a post-authorization prospective national population-based surveillance using the well-established ANSM pharmacovigilance system.^28^^,^^29^ This network is based on 31 regional pharmacovigilance centers, which cover all the French territory, and is coordinated by ANSM since 1973.^28^ All ambulatory or hospital-based health practitioners throughout France or patients that observe a suspected adverse drug reaction (ADR) report the event to the regional center via a secure platform.^28^ Reporting of all ADRs, independently of the seriousness or expectedness, is compulsory for health practitioners. All reports undergo a pharmacological, clinical and biological assessment process by a trained assessor of the Regional Center. All reports are then registered in the national computerized pharmacovigilance database, centralized at ANSM, to allow anonymized case reviewing at a national level by ANSM and experts in the field, drug causality assessment, and to recommend specific measures if required.^29^ The detailed methodology of this French pharmacovigilance system has been previously published.^28^^,^^29^

As part of the national COVID-19 vaccination campaign, ANSM had put in place a specific reinforced passive and active surveillance system to provide real-time monitoring of COVID-19 vaccines ADRs at a national level.^23^^,^^30^ This is part of the risk management plan coordinated by the European Medicines Agency (EMA). The objectives are to carry out a continuous assessment of the safety of vaccines COVID-19 vaccines in order to confirm their safety or to quickly take the relevant measures, and to allow the Health Ministry to adapt the vaccination strategy, if necessary. For each marketed COVID-19 vaccine, two to five regional pharmacovigilance centers have been designated to gather and assess on a daily basis all adverse drug reactions reported following immunization. An expert of the organ involved is solicited to analyze all reported cases every week, to identify atypical and/or serious patterns leading to potential safety signals.^23^ Then a weekly meeting involving ANSM and all regional pharmacovigilance centers is organized to discuss the expert pharmacovigilance reports, potential safety signals, and new data from the literature, in order to confirm or not safety signals.^23^^,^^30^ A complete report including the synopsis of these meeting are published by ANSM every two weeks.^23^^,^^30^ If a national safety signal is validated, appropriate measures are issued in relation with European Medicine Agency to prevent or reduce the likelihood of the risk occurring in vaccinated people. The detailed methodology of this specific COVID-19 vaccine monitoring is available elsewhere.^23^^,^^30^

### Cases review to assess WHO criteria for MIS-C

All paediatric cases of inflammatory syndrome, fever > 3 days, shock, or acute organ dysfunction without any obvious cause, occurring any time after COVID-19 mRNA vaccine injection in children under 18 years of age in France reported to the surveillance system from June 15^th^, 2021, to January 1^st^, 2022, were eligible. Each case was reviewed by a multidisciplinary committee, with experts in pediatric immunization, pediatric infectious diseases, pediatric rheumatology, immunology and internal medicine, pediatric intensive care, pediatric cardiology, and expert pharmacologists from pharmacovigilance centers. All these experts were involved in MIS-C surveillance and management in France as part of the French MIS-C consortium, and developed specific expertise in this field.^5^^,^^31^^,^^32^ Medical records were obtained for all cases to accurately assess if cases fulfilled WHO criteria for MIS-C, with COVID-19 mRNA vaccine exposure replacing SARS-CoV-2 exposure criteria, as indicated by recent Brighton Collaboration definition guidelines.^33^ Cases were included after reviewing if they fulfilled WHO MIS-C criteria, with a delay between the last vaccine administration and disease onset < 2 months, based on available data from the literature regarding the delay between SARS-CoV-2 infection and MIS-C onset.^4^, ^5^, ^6^ An important part of the vaccine causality assessment relied on identifying other potential causes for the event.^33^^,^^34^ For hyper-inflammatory syndrome, extensive investigation of previous exposure to SARS-CoV-2 over the past two months was critical, and relied on investigating history of documented infection, and performing nasopharyngeal SARS-CoV-2 Polymerase chain reaction (PCR) and anti-Nucleocapsid (anti-N) serology.^33^^,^^35^

### National immunization program

BNT162b2 COVID-19 mRNA vaccine have been introduced for 12–17-year-old children on June 15th, 2021 in France.^36^ It has been followed by mRNA-1273 approval for same age children on July, 27th, 2021.^36^ A higher risk of myocarditis or pericarditis has been suggested following mRNA-1273 compared to BNT162b2 in adults younger than 30 years old.^37^ This has led French authorities to prioritize BNT162b2 for 12–17-year-old children immunization. Thus, by January 1^st^, 2022, the large majority of vaccinated 12–17-year-old children received BNT162b2 (>95%).^32^

### Outcome measure

The main outcome was the national reporting rate of hyper-inflammatory syndrome following COVID-19 mRNA vaccine per 1,000,000 doses in 12-17-year-old children in France. To calculate national reporting rate, we used as a denominator the total number of COVID-19 mRNA vaccine dose administered in 12-17-year-old children over the study period (available at https://solidarites-sante.gouv.fr/grands-dossiers/vaccin-covid-19/article/le-tableau-de-bord-de-la-vaccination). We also analyzed the reporting rate of hyper-inflammatory syndrome following COVID-19 mRNA vaccine per 1,000,000 12-17-year-old vaccinated children in France.

Then, we estimated in the same age-group, in the same population, the rate of post-SARS-CoV-2 MIS-C cases per 1,000,000 infections in 12-17-year-old children in France for comparison.

Secondary outcomes were the reporting rate of hyper-inflammatory syndrome following first and second injections of COVID-19 mRNA vaccine in 12-17-year-old children in France, reporting rate by sex, and comparison of clinical features of hyper-inflammatory syndrome following COVID-19 mRNA vaccine versus post-SARS-CoV-2 MIS-C cases.

### MIS-C following SARS-CoV-2 infection surveillance system

To estimate the rate of post-SARS-CoV-2 MIS-C cases per 1,000,000 infections in 12-17-year-old children in France, we used data from the French COVID-19 Pediatric Inflammation Consortium, coordinated by Public health France.^5^^,^^31^^,^^32^ As previously published, since April 2020, all suspected MIS-C cases in France were mandatorily reported to Public health France. Each suspected case was then assessed following WHO criteria for MIS-C.^5^^,^^31^^,^^32^ Furthermore, Public health France also conducted seroprevalence studies that allowed estimating the proportion of 12-17-year-old old children infected by SARS-CoV-2 since the beginning of the pandemic.^38^ Thus, to estimate the rate of post-SARS-CoV-2 MIS-C cases per 1,000,000 infections, we used as a numerator the number of confirmed 12-17-year-old MIS-C cases reported to Public health France since the start of the pandemic, and as a denominator the estimated number of 12-17-year-old French children infected by SARS-CoV-2 since the start of the pandemic, based on Public health France estimations.^38^ To avoid any bias in MIS-C rate estimation due to vaccine implementation,^32^ we restricted this analysis to the pre-vaccine period, i.e. from the start of the pandemic to June 15^th^, 2021.

This surveillance system also collected clinico-biological and short term outcome data of MIS-C cases that fulfilled WHO criteria.^5^^,^^31^^,^^32^ Thus, to compare the clinical presentation of hyper-inflammatory syndrome following COVID-19 mRNA vaccine to post-SARS-CoV-2 MIS-C cases in the same population, we included all unvaccinated 12-17-year-old MIS-C cases fulfilling WHO criteria with available clinical files as same-age and same-population comparator group.

### Statistical analysis

We describe patient characteristics with numbers (percentages) for categorical variables and median (interquartile range [IQR]) for quantitative variables. We compared clinical and biological characteristics using non-parametric Fisher's exact test for categorical variables and Mann-Whitney U test for quantitative variables. A two-sided p-value <0.05 was considered statistically significant. Reporting rate of hyper-inflammatory syndrome was expressed as cases per 1,000,000 vaccine injections with 95% CIs. All statistical analyses involved using R v3.6.1 (http://www.R-project.org).

### Ethics

For the pharmacovigilance surveillance system, this study was performed according to the authorization from the National Commission on Informatics and Liberty (CNIL) n° 2014-302 for the national pharmacovigilance database done by ANSM. For the MIS-C following SARS-CoV-2 infection surveillance system, the study was approved by the INSERM ethics committee for evaluation (IRB00003888). A written information form validated by the ethics committee was given to all participants. Oral consent was obtained from study participants; no family members or participants refused to participate.

## Results

From June 15th, 2021 to January 1st, 2022, 8,113,058 COVID-19 mRNA vaccine doses were administered to 4,079,234 12-17-year-old children in France (including 4,079,234 first injections, 3,905,636 second injections, and 128,188 third injections). Over this period, 2,028 adverse drug reactions related to COVID-19 mRNA vaccine have been reported to the pharmacovigilance centers in 12-17-year-old children. Among them, 102 cases of myocarditis or pericarditis (including 78 (76%) males), and 12 cases of hyper-inflammatory syndrome were reported. All hyper-inflammatory syndrome cases fulfilled WHO criteria for MIS-C (Table 1). Ten cases involved BNT162b2 vaccine and 2 cases involved mRNA-1273. Six cases occurred following the first injection, and 6 following the second injection. As for myocarditis and pericarditis, a male predominance was also observed in these hyper-inflammatory syndromes (10/12 cases, 83%). The delay between last injection and disease onset ranged from 1 day to 42 days.

**Table 1:** Characteristics of children with hyper inflammatory syndrome following COVID-19 mRNA in France.

Case	1	2	3	4	5	6	7	8
Age (y)	12	15	16	13	12	13	12	12
Sex	Male	Male	Male	Male	Male	Male	Female	Male
Comorbidity	No	No	No	No	Type 1 diabetes	Osteochondritis	Leukemia	No
Overweight	No	No	No	No	No	Yes	No	No
Symptom onset	November 2021	August 2021	September 2021	October 2021	October 2021	July 2021	September 2021	December 2021
Number of COVID-19 mRNA injection	2	1	1	2	1	1	1	2
COVID-19 mRNA vaccine	BNT162b2	BNT162b2	BNT162b2	BNT162b2	BNT162b2	BNT162b2	BNT162b2	BNT162b2
Delay from COVID-19 mRNA last injection to symptoms onset	26 days (50 days from first injection)	6 days	6 days	2 days (24 days from first injection)	4 days	20 days	19 days	42 days (72 days from first injection)
MIS-C WHO criteria£	Yes	Yes	Yes	Yes	Yes	Yes	Yes	Yes
Details of MIS-C WHO criteria	Fever > 3 days, Mucocutaneous involvement, Cardiac involvement, Elevated markers of inflammation, No other obvious microbial cause	Fever > 3 days, Shock, Cardiac involvement, Coagulopathy, Acute gastrointestinal symptoms, Elevated markers of inflammation, No other obvious microbial cause	Fever > 3 days, Mucocutaneous involvement, Shock, Cardiac involvement, Coagulopathy, Acute gastrointestinal symptoms, Elevated markers of inflammation, No other obvious microbial cause	Fever > 3 days, Cardiac involvement, Coagulopathy, Acute gastrointestinal symptoms, Elevated markers of inflammation, No other obvious microbial cause	Fever > 3 days, Mucocutaneous involvement, Shock, Cardiac involvement, Acute gastrointestinal symptoms, Elevated markers of inflammation, No other obvious microbial cause	Fever > 3 days, mucocutaneous involvement, Cardiac involvement, Elevated markers of inflammation, No other obvious microbial cause	Fever > 3 days, Coagulopathy, Acute gastrointestinal symptoms, Elevated markers of inflammation, No other obvious microbial cause	Fever > 3 days, Coagulopathy, Cardiac involvement, Acute gastrointestinal symptoms, Elevated markers of inflammation, No other obvious microbial cause
Other manifestations	Lymphopenia, Cervical lymphadenopathy	Acute renal failure, proteinuria, Cytolytic hepatitis, Neurological involvement, Polyserositis, Hypereosinophilia.	Lymphopenia, Coronary dilation.	Acute renal failure, Cytolytic hepatitis, Pyelitis, Ileo-colitis.	Cervical lymphadenopathy, Hypereosinophilia, Acute generalized exanthematous pustulosis.	Lymphopenia.	Macrophage activation syndrome, Cytolytic hepatitis.	Lymphopenia, Cervical lymphadenopathy Cytolytic hepatitis.
*Biological features*								
CRP, mg/L	250	300	257	228	70	97	49	167
Ferritinemia (µg/L)	527	195	600	Not performed	309	9185	25 020	430
Hemoglobin (g/dL)	12.6	11.6	10.6	14.6	NA	NA	15.6	12.3
Leucocytes (/mm3)	16 500	11 130	16 600	4 050	NA	12 800	1 250	10 000
Neutrophils (/mm3)	15 500	4 340	13 000	Not performed	NA	9 700	1 040	9 400
Lymphocytes (/mm3)	520	3 560	900	Not performed	NA	890	100	920
Eosinophils (/mm3)	20	2 000	310	Not performed	1 170	NA	0	220
Platelets (/mm3)	225 000	472 000	278 000	321 000	204 000	350 000	27 000	230 000
*SARS-CoV-2 infection documentation*								
Past history of SARS-CoV-2 infection	No	No	No	No	Yes (documented SARS-CoV-2 infection 7 months before)	No	No	No
Nasopharyngeal SARS-CoV-2 PCR	Negative	Negative	Negative	Negative	Negative	Negative	Negative	Negative
SARS-CoV-2 antibody	Anti-Spike: positive Anti-N: negative	Anti-Spike: positive Anti-N: negative	Anti-Spike: positive Anti-N: positive	Anti-Spike: positive Anti-N: positive	Anti-Spike: positive Anti-N: negative	Anti-Spike: positive Anti-N: negative	Anti-Spike: negative Anti-N: negative	Anti-Spike: positive Anti-N: limit of significance*
Delay from first vaccine injection to SARS-CoV-2 antibody testing	51 days	15 days	14 days	34 days	7 days	33 days	23 days	85 days
Specific therapy	IVIG+ steroids	None	IVIG+ steroids	IVIG+ steroids	IVIG+ steroids followed by steroid pulse	IVIG+ steroids	Steroids	IVIG+ steroids
PICU transfer	Yes	Yes	No	No	Yes	No	No	No
LVEF ≤ 55%	Yes (55%)	No	No	Yes (40%)	Yes	No	No	No
Hemodynamic support	No	Yes	No	No	Yes	No	No	No
Outcome	Favorable	Favorable	Favorable	Favorable	Favorable	Favorable	Favorable	Favorable
Hospital length of stay (days)	6	7	13	7	9	8	7	9

Case	9	10	11	12				
Age (y)	12	12	12	13				
Sex	Male	Male	Female	Male				
Comorbidity	No	No	Transient thyroiditis	No				
Overweight	No	No	Yes	No				
Symptom onset	December 2021	September 2021	October 2021	October 2021				
Number of COVID-19 mRNA injection	2	1	2	2				
COVID-19 mRNA vaccine	BNT162b2	BNT162b2	mRNA-1273	mRNA-1273				
Delay from COVID-19 mRNA last injection to symptoms onset	7 days (28 days from first injection)	16 days	24 days (48 days from first injection)	1 day (21 days from first injection)				
MIS-C WHO criteria£	Yes	Yes	Yes	Yes				
Details of MIS-C WHO criteria	Fever > 3 days, mucocutaneous involvement,Cardiac involvement, Acute gastrointestinal symptoms, Elevated markers of inflammation, No other obvious microbial cause	Fever > 3 days, Shock, Cardiac involvement, Acute gastrointestinal symptoms, Elevated markers of inflammation, No other obvious microbial cause	Fever > 3 days, mucocutaneous involvement, Shock, Cardiac involvement, Coagulopathy, Acute gastrointestinal symptoms, Elevated markers of inflammation, No other obvious microbial cause	Fever > 3 days, Mucocutaneous involvement, Coagulopathy, Acute gastrointestinal symptoms, Elevated markers of inflammation, No other obvious microbial cause				
Other manifestations	Lymphopenia, Ileitis.	Polyserositis,	Cytolytic hepatitis, Hepato-splenomegaly Lymphopenia,	Cytolytic hepatitis, Lymphopenia, Poly-arthralgia Neurological involvement, myalgia				
*Biological features*								
CRP, mg/L	186	102	150	109				
Ferritinemia (µg/L)	Not performed	Not performed	Not performed	Not performed				
Hemoglobin (g/dL)	11.5	10.3	11.8	13.4				
Leucocytes (/mm3)	8 690	12 000	10 400	8 000				
Neutrophils (/mm3)	7 600	10 000	9 560	6 730				
Lymphocytes (/mm3)	540	NA	580	510				
Eosinophils (/mm3)	10	NA	NA	320				
Platelets (/mm3)	113 000	600 000	220 000	192 000				
*SARS-CoV-2 infection documentation*								
Past history of SARS-CoV-2 infection	No	No	No	No				
Nasopharyngeal SARS-CoV-2 PCR	Negative	Negative	Negative	Negative				
SARS-CoV-2 antibody	Anti-Spike: positive Anti-N: positive	Anti-Spike: positive Anti-N: positive	Anti-Spike: positive Anti-N: negative	Anti-Spike: not performed Anti-N: negative				
Delay from first vaccine injection to SARS-CoV-2 antibody testing	32 days	22 days	50 days	24 days				
Specific therapy	Steroid pulse	IVIG+ steroids	IVIG+ steroids	Steroids				
PICU transfer	No	Yes	No	No				
LVEF ≤ 55%	Yes (40%)	Yes (35%)	Yes (50%)					
Hemodynamic support	No	Yes	No	No				
Outcome	Favorable	Favorable	Favorable	Favorable				
Hospital length of stay (days)	7	9	5	5				

Biological parameters were at admission, except for CRP, which is the maximal value during the hospitalization.

Abbreviations: PCR: polymerase chain reaction. Anti-N: anti nucleocapsid. NA: missing data.

⁎ antibody titer: 1.2 (Norms of the laboratory: positive: ≥ 1.68, negative: <0.49, limit: 0.49 ≤ x < 1.68).

£ MIS-C criteria with COVID-19 mRNA vaccine exposure replacing SARS-CoV-2 exposure.

### Investigation of previous SARS-CoV-2 infection

All 12 cases had complete data for history of documented infection, nasopharyngeal SARS-CoV-2 PCR, and anti-N serology. In one case, a previous infection 7 months before disease onset was reported, but was too far apart to be considered as linked to the disease. All children had negative SARS-CoV-2 PCR, and four children had a positive anti-N serology (Table 1). In 8 cases, no evidence of previous SARS-CoV-2 infection was found (details Table 1). The clinical and biological details of cases depending on SARS-CoV-2 anti Nucleocapsid serology is provided Table S1.

### National reporting rate of hyper-inflammatory syndrome following mRNA vaccine

Considering all 12 cases of hyper-inflammatory syndrome, a national reporting rate of 1.5 (95% CI [0.8; 2.6]) per 1,000,000 mRNA vaccine doses administered in 12-17-year-old children was observed. Excluding cases for which evidence of previous SARS-CoV-2 infection has been found, the reporting rate was reduced to 1.0 (95% CI [0.4; 1.9]) per 1,000,000 mRNA vaccine doses administered. This reporting rate was similar following the first and the second mRNA vaccine injections (Table 2). The reporting rate was significantly higher for males compared to females (2.4 [1.1; 4.5] versus 0.5 [0.1; 1.8] per 1,000,000 doses, respectively, p=0.039).

**Table 2:** Rate of hyper-inflammatory syndrome following COVID-19 mRNA vaccine compared to MIS-C post SARS-CoV-2 infection in 12-17-year-old children in France.

A) Reporting rate of hyper-inflammatory syndrome following COVID-19 mRNA vaccine in 12-17-year-old children			
	Number of injected doses	Number of hyper-inflammatory syndrome	Reporting rate per 1,000,000 doses
Overall vaccination	8,113,058	12	1.5 [0.8; 2.6]
Excluding cases for which evidence of previous SARS-CoV-2 infection has been found	8,113,058	8	1.0 [0.4; 1.9]
First COVID-19 mRNA injection	4,079,234	6	1.5 [0.5; 3.2]
Second COVID-19 mRNA injection	3,905,636	6	1.5 [0.6; 3.3]
*Vaccination by sex*			
Males	4,126,275	10	2.4 [1.1; 4.5]
Females	3,986,783	2	0.5 [0.1; 1.8]
Rate per 1,000,000 of vaccinated children	Number of vaccinated children: 4,079,234	12	2.9 [1.5; 5.1]
			

B) Reporting rate of myocarditis or pericarditis following COVID-19 mRNA vaccine in 12-17-year-old children			
	Number of injected doses	Number of myocarditis or pericarditis	Reporting rate per 1,000,000 doses
Overall vaccination	8,113,058	102	12.6 [10.3; 15.3]
*Vaccination by sex*			
Males	4,126,275	78	19.0 [14.9; 23.6]
Females	3,986,783	24	6.0 [3.9; 9.0]
Rate per 1,000,000 of vaccinated children	Number of vaccinated children: 4,079,234	102	25.0 [20.4; 30.4]
			

C) Rate of MIS-C following SARS-CoV-2 infection in 12-17-year-old children			
	Estimated number of infected children	Number of MIS-C	Rate per 1,000,000 of infections
	1,147,150	130	113.3 [94.7; 134.6]
*MIS-C by sex*			
Males	587,086	74	126.0 [99.0; 158.2]
Females	560,064	56	100.0 [75.5; 129.8]

Abbreviations: MIS-C: multisystem inflammatory syndrome in children.

The reporting rate per 1,000,000 12-17-years old vaccinated children was 2.9 [1.5; 5.1]. The reporting rate of myocarditis or pericarditis per 1,000,000 mRNA vaccine doses administered in 12-17-year-old children is provided Table 2B.

As a comparator, 130 post-SARS-CoV-2 MIS-C cases occurred in 12-17-year-old children, for 1,148,299 same-age children infected by SARS-CoV-2, leading to a MIS-C rate of 113.3 [94.7; 134.6] per 1,000,000 12-17-year-old infected children (Table 2).

### Clinical features of hyper-inflammatory syndrome following COVID-19 mRNA vaccine compared to post-SARS-CoV-2 MIS-C cases

The detailed clinical presentation of children with hyper-inflammatory syndrome following COVID-19 mRNA vaccine is provided Table 1. Median age was 12.0 years (IQR [12.0; 13.0]), 10/12 (83%) children were male and 3/12 had comorbidities (one type 1 diabetes, one osteochondritis with overweight, and one leukemia in remission). The most frequent clinical features were cardiac involvement (10/12, 83%, including 9 elevated cardiac enzymes, 5 acute left ventricular ejection fraction decrease ≤ 55%, 4 pericarditis, 1 transient coronary dilation and 1 myocarditis), gastrointestinal symptoms (10/12, 83%), coagulopathy (7/12, 58%), mucocutaneous involvement (7/12, 58%), cytolytic hepatitis (6/12, 50%) and shock (5/12, 42%). Macrophage activation syndrome was identified in one case. For comparison, among 199 children with post-SARS-CoV-2 MIS-C, 108 (54%) were male (p=0.071), cardiac involvement was found in 63% (p=0.219), and hepatic involvement was significantly less frequent (36/199 cases, 18%, p=0.016, Table 3).

**Table 3:** Comparison of clinic-biological features of hyper-inflammatory syndrome following COVID-19 mRNA vaccine and MIS-C post SARS-CoV-2 infection in France.

	Hyper-inflammatory syndrome following COVID-19 mRNA vaccine (N=12)	MIS-C post SARS-CoV-2 infection (N=199)	P value
Clinical characteristics			
Sex ratio (F/M)	0.2	0.84	0.071
Age	12.0 [12.0; 13.0]	8.7 [4.8; 12.1]	
*Organ involvement following MIS-C WHO definition*			
Mucocutaneous involvement	7 (58%)	159 (80%)	0.137
Shock	5 (42%)	100 (50%)	0.557
Cardiac involvement	10 (83%)	125 (63%)	0.219
Including LVEF ≤ 55%	5 (42%)	52 (26%)	0.313
Coagulopathy	7 (58%)	82 (41%)	0.367
Digestive symptoms	10 (83%)	178 (89%)	0.833
*Other organ involvement*			
Cytolytic hepatitis	6 (50%)	36 (18%)	0.016
Lymphadenopathy	3 (25%)	39 (20%)	0.721
Renal failure	2 (17%)	31 (16%)	1.0
Neurological involvement	2 (17%)	65 (33%)	0.346
Biological features			
Maximal CRP, mg/L	158.5 [100.8; 233.5]	233.0 [153.5; 310.8]	0.019
Ferritinemia, (µg/L)	527.0 [369.5; 4892.5]	390.0 [206.0; 748.5]	0.228
Hemoglobin, g/dL	12.1 [11.5; 13.2]	11.0 [10.1; 11.0]	0.017
Leucocytes, /mm3	10 400 [8 345; 12 400]	9 945 [7 000; 14 155]	0.761
Neutrophils, /mm3	9 480 [6 947; 9 925]	8 090 [5 245; 12 065]	0.823
Lymphocytes, /mm3	580 [520; 900]	1 000 [690; 1 805]	0.069
Eosinophils, /mm3	265 [175; 533]	100 [5; 295]	0.435
Platelets, /mm3	227 500 [201 000; 328 000]	186 000 [142 000; 277 500]	0.160
Short term outcomes			
PICU transfer	4 (33%)	143 (72%)	0.008
Hemodynamic support	3 (25%)	86 (43%)	0.24
Hospital length of stay	7.0 [6.8; 9.0]	8.0 [6.0; 11.0]	0.719

Categorical variables are described with numbers (percentages) and quantitative variables are described with median (IQR). Biological parameters were at admission, except for CRP, which is the maximal value during the hospitalization.

Abbreviations: MIS-C: multisystem inflammatory syndrome in children.

Some biological parameters also differed between post-vaccine hyper-inflammatory syndrome and post-SARS-CoV-2 MIS-C, including inflammatory parameters (median CRP level 159 101; 234] vs 233 [154; 311], respectively, p=0.019), and blood cell count (median hemoglobin 12.1 [11.5; 13.2] vs 11.0 [10.1; 11.0], respectively, p=0.017, Table 3). Of note, 2/12 (17%) children with post-vaccine hyper-inflammatory syndrome had a transient hypereosinophilia (compared to 9/199 (5%) in post-SARS-CoV-2 MIS-C). These two children were explored for TRBV11-2/Vb21.3 T cell receptor (TCR) expansion by flowcytometry, none of them had an expansion of this repertory, while it was present in 75% of post-SARS-CoV-2 MIS-C patients.^12^ The other patients were not tested for TCR repertoire expression.

### Specific therapy and short-term outcomes

Short term outcomes seemed also less severe for post-vaccine hyper-inflammatory syndrome, with a lower rate of PICU transfer (4/12, 33%), compared to 143/199 (72%) for post-SARS-CoV-2 MIS-C (p=0.008, Table 3). Eight children were treated by an administration of intravenous immunoglobulins plus methylprednisolone, of whom one received a subsequent 10mg/kg/day methylprednisolone pulse. Three children received methylprednisolone alone, and one did not receive specific immunomodulator agent. All children fully recovered at the time of discharge. Median length of hospital stay was 7 days (IQR [7; 9]).

## Discussion

This post-authorization population-based pharmacovigilance study assessed the risk of hyper-inflammatory syndrome following COVID-19 mRNA vaccine in 12-17-year-old children, and compared this rate to post-SARS-CoV-2 MIS-C. We found that this entity was observed with a reporting rate of 1.5 (95% CI [0.8; 2.6]) per 1,000,000 doses in this population, i.e. 2.9 (95%CI [1.5; 5.1]) per 1,000,000 12–17-year-old vaccinated children. A recent surveillance investigation in the USA also reported cases of hyper-inflammatory syndrome in children who received COVID-19 vaccine, with a similar reporting rate (1.0 per 1,000,000 individuals receiving at least one dose).^39^ In most cases in our study, no evidence of previous SARS-CoV-2 infection was observed, suggesting a potential link between this entity and COVID-19 mRNA vaccine. This rare serious adverse event should be put in balance with the rate of post-SARS-CoV-2 MIS-C in the same age group in the same population, which was much higher. Two recent studies highlighted that COVID-19 mRNA vaccine may significantly reduce the incidence of post-SARS-CoV-2 MIS-C.^32^^,^^40^ Taken together, these findings suggest that the benefit-risk balance of COVID-19 mRNA vaccine is largely in favor of the vaccination in this age group, in a context of active circulation of SARS-CoV-2.

An important issue is to delineate the clinical spectrum of this entity, which may have overlap with several other diseases. First, an increased risk of myocarditis has been reported following COVID-19 mRNA vaccines, especially in young men.^19^^,^^41^ These myocarditis mainly occurred after the second dose of vaccine, few days after the injection, and were rapidly resolutive in most cases.^19^^,^^41^ The male predominance was also observed in post vaccine myocarditis occurring in 12-17 years old French population (78/102 cases, 76%). These characteristics echo to the male predominance observed in our study among children with hyper-inflammatory syndrome following COVID-19 mRNA vaccine (10/12 cases, 83%), as well as the important rate of cardiac involvement (10/12 cases, 83%). On the other hand, myocarditis were classically afebrile, with low inflammatory parameters, and were a mono-organ involvement.^41^ These major clinical differences may allow distinguishing these two entities, that however could possibly represent a continuum. Second, all cases of hyper-inflammatory syndrome following COVID-19 mRNA vaccine fulfilled WHO definition for MIS-C. Indeed, the prolonged hyper-inflammatory state, the multi-organ involvement and the severity of the disease are principal features of these two entities,^8^ indicating at least a major overlap. However, if statistical comparison between these two diseases was limited by the low number of cases, and should be interpreted with caution, our findings suggest that post-SARS-CoV-2 MIS-C may have still higher inflammatory parameters, and more cytopenia. This may be in line with the significantly lower rate of PICU transfer (33% vs 72%) for hyper-inflammatory syndrome following COVID-19 mRNA vaccine cases, which might reflect a less severe immune storm and disease course. Notably, a 4-week delay has been observed in the context of MIS-C following SARS-CoV-2 infection.^42^, ^43^, ^44^ Here, the delay from first antigen exposure to hyper-inflammatory syndrome occurred within a week in 3 patients and after 4–12 weeks in the 9 others. In the cases with early reaction, a hypereosinophilia was seen in 2 patients, a feature not seen in classical MIS-C. This observation might reflect immunoallergic reaction distinct from the superantigenic like features of MIS-C. Expansion of Vb21.3 expressing T cells is a hallmark of the MIS-C and can be easily assessed by flowcytometry.^12^ By contrast, the two children with post-vaccination hyper-inflammatory syndrome had no expansion of this T cell subset. Taken together, these clinical and immunological divergences may imply distinct underlying pathways and further studies are required to expand this finding. Third, a recent study coordinated by the CDC reported cases of multisystem inflammatory system in adults (MIS-A) in USA, in vaccinated and unvaccinated adults.^35^ Twenty cases were identified, of whom seven were vaccinated. However, all of them had a documented previous exposure to SARS-CoV-2, questioning the direct causal role of mRNA vaccines in these manifestations, and diverging with the pediatric syndrome reported here, with only 4/12 patients presenting a seropositivity to N antigen. The issue of delineating these different entities underline the need to extensively investigate cases of hyper-inflammatory syndromes following mRNA vaccines, especially by performing anti-S and anti-N serology, along with exploration for TRBV11-2/Vb21.3 expansion.

Because this hyper-inflammatory syndrome following COVID-19 mRNA vaccine was severe with acute multi-organ dysfunction, therapeutic aspects are of major interest. In this cohort, most children were treated by an association of immunoglobulins plus methylprednisolone, following MIS-C therapeutic protocols.^31^ Only one child treated with this combination required a therapeutic escalation, and received a methylprednisolone pulse (10mg/kg/day). All children fully recovered. If sample size precludes any definitive conclusion, these findings suggest that the association of immunoglobulins plus methylprednisolone may be a suitable approach while awaiting more evidence regarding these treatments.

Several limitations should be discussed. First, a causal association between COVID-19 mRNA vaccines and hyper-inflammatory syndrome could not be asserted. Indeed, despite extensive investigation for previous SARS-CoV-2 infection in all cases, pauci or asymptomatic SARS-CoV-2 infections are frequent in children, and may not have been documented. Furthermore, false negatives can be observed for anti-Nucleocapsid serology.^45^ Thus, we cannot exclude that some of the cases reported here could be related to undiagnosed SARS-CoV-2 infections. Furthermore, an important step in assessing vaccine related adverse events is to compare them to the rate of the disease in unvaccinated populations, as it was done for myocarditis. Because this entity has not been previously described in healthy populations, we could not have a control population to estimate the expected incidence of this disease in unvaccinated children. In addition, as this event is very rare, large population-based pharmacovigilance studies from other countries are required to precise the role of COVID-19 mRNA vaccine in this syndrome.^17^ Second, we cannot rule out under reporting of adverse drug events in our population, which may have biased the estimated rate of hyper-inflammatory syndrome. However, following the implementation of COVID-19 mRNA vaccine, a major effort has been made by all pharmacovigilance centers to publicize that the reporting of any suspected adverse drug reaction following these vaccines was mandatory.^28^ The impressive number of suspected adverse drug reaction reports (>80,000 between January 2021 and January 2022 in France) suggest that underreporting may have been very rare, especially for serious adverse drug reactions.^28^ In France, national guidelines recommend any suspected MIS-C case to be hospitalized, making the possibility of underreporting due to ambulatory care of hyper inflammatory syndrome unlikely .Third, given the very low proportion of 12-17-year-old children vaccinated by mRNA-1273 (<5%), we could not provide an accurate estimation of the risk of hyper-inflammatory syndromes according to COVID-19 mRNA vaccine type. Further studies are required to explore if this risk differ between BNT162b2 and mRNA-1273. Fourth, because mRNA vaccines were only recommended to 12–17-year-old children in France until December 2021, we could not explore the risk of hyper-inflammatory syndrome in younger children.

## Conclusion

In this study, we identified cases of hyper-inflammatory syndrome following COVID-19 mRNA vaccines in 12-17-year-old children in France, with multisystemic involvement. This syndrome was very rare, and its reporting rate, in comparison with the rate of MIS-C following SARS-CoV-2 infection in the same age-group, largely supports the vaccination in a context of an important circulation of SARS-CoV-2. This syndrome shared many clinical features with post-SARS-CoV-2 MIS-C, but some clinical, immunological and short-term outcomes divergences call for further studies to explore its specific pathway.

## Contributors

NO, HB, FA, and AB made substantial contributions to the conception or design of the work. NO and AB drafted the manuscript. DA, NO, HB, CLeb, CLev, FS, EJ, BBM, CO, SR, VH, IKP, ND, LDP, XI, CGLG, FA and AB were involved in the acquisition, analysis, or interpretation of data. All authors provided critical revision of the manuscript for important intellectual content. NO and HB had full access to all data in the study and take responsibility for the integrity of the data and the accuracy of the data analysis.

Funding/Support: NO was supported by the 2021 ESPID (European Society for Pediatric Infectious Diseases) Fellowship Award. The French Covid-19 Paediatric Inflammation Consortium received an unrestricted grant from the Square Foundation (Grandir–Fonds de Solidarité Pour L'enfance).

Data availability: Data are available upon reasonable request to NO (naim.ouldali@aphp.fr).

Role of the Funder/Sponsor: The funders had no role in the design or conduct of the study, collection management, analysis, or interpretation of the data; preparation, review, or approval of the manuscript; or the decision to submit the manuscript for publication.

Additional Contributions : We are grateful to Agence Nationale de Sécurité du Médicament et des produits de santé (ANSM), Santé Publique France, Société Française de Pédiatrie, Groupe de Pédiatrie Générale, Groupe de Pathologie Infectieuse Pédiatrique, Groupe Francophone de Réanimation et d'Urgences Pédiatriques, Société Française de Cardiologie, Filiale de Cardiologie Pédiatrique et Congénitale, Société Francophone Dédiée à L’étude des Maladies Inflammatoires Pédiatriques, the PANDOR study group and Filière de Santé des Maladies Auto-immunes et Auto-inflammatoires Rares for their participation in the French Covid-19 Paediatric Inflammation Consortium study. We thank Youssef Shaim, Laurence Baril, Mehdi Benkebil, Samuel Crommelynck, Baptiste Jacquot from ANSM, Daniel Levy-Bruhl, from Santé Publique France, Agence Nationale de Santé Publique, Saint-Maurice; Murielle Herasse, from Filière de Santé Des Maladies Auto-immunes et Auto-inflammatoires Rares (FAI2R), Lyon, France; and David Skurnik, PhD, from INSERM U1151-Equipe 11. We are grateful to every microbiological laboratory staff member who performed severe acute respiratory syndrome coronavirus 2 reverse transcriptase–polymerase chain reaction and antibody testing in each center. None of the persons listed here received compensation for their role in the study.

## Declaration of Interests

Dr N. Ouldali reports travel grants from GSK, Pfizer, and Sanofi. Dr C. Levy reported receiving grants from Pfizer and personal fees from Pfizer and Merck. Pr R. Cohen reported receiving personal fees from GlaxoSmithKline, Pfizer, Sanofi, and Merck Sharp & Dohme. Dr V. Hentgen reports travel grants from Novartis and Sobi. Pr F. Angoulvant reports receiving personal fees from MSD, Astrazeneca, Sanofi and Pfizer. Pr A. Belot reports receiving personal fees from GlaxoSmithKline, Novartis, Sobi and Pfizer, and grants from Merck Serono and Boehringer Ingelheim. All other authors have no potential conflicts of interest to disclose.

## Appendix Supplementary materials

Image, application 1
