# Supplementary material for: Hyper inflammatory syndrome following COVID-19 mRNA vaccine in children: A national post-authorization pharmacovigilance study
Source: Lancet Reg Health Eur. 2022 Apr 29;17:100393. doi: 10.1016/j.lanepe.2022.100393 (PMC9051933; doi:10.1016/j.lanepe.2022.100393)
Supplement: Supplementary file 1 [file mmc1.docx]

**Hyper inflammatory syndrome following COVID-19 mRNA vaccine in children: a national post-authorization pharmacovigilance study**

**Authors:** Naïm Ouldali, M.D., Ph.D., Haleh Bagheri, Pharm.D., Ph.D, Francesco Salvo, M.D., Ph.D., Denise Antona, M.D., Antoine Pariente, M.D., Ph.D., Claire Leblanc, M.D., Martine Tebacher, M.D., Ph.D., Joëlle Micallef, M.D., Ph.D., Corinne Levy, M.D., Robert Cohen, M.D., Etienne Javouhey, M.D., Ph.D., Brigitte Bader-Meunier, M.D., Caroline Ovaert, M.D., Ph.D., Sylvain Renolleau, M.D., Ph.D., Veronique Hentgen, M.D., Ph.D., Isabelle Kone-Paut, M.D., Nina Deschamps, M.D., Loïc De Pontual, M.D., Ph.D., Xavier Iriart, M.D., Christelle Gras-Le Guen, M.D., Ph.D., François Angoulvant, M.D., Ph.D., Alexandre Belot, M.D., Ph.D, and the “French Covid-19 Paediatric Inflammation Consortium”^£^ and the “French Pharmacovigilance network”

**APPENDICES:**

Table S1. Characteristics of children with hyper inflammatory syndrome following COVID-19 mRNA in France depending on SARS-CoV-2 anti Nucleocapsid serology results.

Appendix 1. STROBE checklist.

Appendix 2. Members of the French Covid-19 Paediatric Inflammation Consortium and the French Pharmacovigilance network

Table S1. Characteristics of children with hyper inflammatory syndrome following COVID-19 mRNA in France depending on SARS-CoV-2 anti Nucleocapsid serology results.

|  | **Negative SARS-CoV-2 anti Nucleocapsid serology (N=8)** | **Positive SARS-CoV-2 anti Nucleocapsid serology (N=4)** |
| --- | --- | --- |
| **Clinical characteristics** |  |  |
| Sex ratio (F/M) | 0.25 | 0 |
| Age | 12.0 [12.0; 13.0] | 12.5 [12.0; 13.8] |
| *Organ involvement following MIS-C WHO definition* |  |  |
| Mucocutaneous involvement | 5 (63%) | 2 (50%) |
| Shock | 3 (38%) | 2 (50%) |
| Cardiac involvement | 6 (75%) | 4 (100%) |
| Including LVEF ≤ 55% | 2 (25%) | 3 (75%) |
| Coagulopathy | 4 (50%) | 3 (75%) |
| Digestive symptoms | 6 (75%) | 4 (100%) |
| *Other organ involvement* |  |  |
| Cytolytic hepatitis | 5 (63%) | 1 (25%) |
| Lymphadenopathy | 3 (38%) | 0 (0%) |
| Renal failure | 1 (13%) | 1 (25%) |
| Neurological involvement | 2 (25%) | 0 (0%) |
| **Biological features** |  |  |
| Maximal CRP, mg/L | 129.5 [90.3; 187.8] | 207.0 [165.0; 235.2] |
| Ferritinemia, (µg/L) | 478.5 [339.2; 7020.5] | 600.0 [600.0; 600.0] |
| Hemoglobin, g/dL | 12.5 [11.9; 13.2] | 11.1 [10.5; 12.3] |
| Leucocytes, /mm3 | 10 400 [9 000; 11 960] | 10 350 [7 530; 13 155] |
| Neutrophils, /mm3 | 9 480 [6 947; 9 925] | 8 090 [5 245; 12 065] |
| Lymphocytes, /mm3 | 580 [515; 905] | 720 [630; 810] |
| Eosinophils, /mm3 | 270 [70; 958] | 160 [85; 235] |
| Platelets, /mm3 | 299 500 [236 800; 390 800] | 222 500 [201 000; 260 000] |
| **Short term outcomes** |  |  |
| PICU transfer | 3 (38%) | 1 (25%) |
| Hemodynamic support | 2 (25%) | 1 (25%) |
| Hospital length of stay | 7.0 [5.8; 8.3] | 8.0 [7.0; 10.0] |

Categorical variables are described with numbers (percentages) and quantitative variables are described with median (IQR). Biological parameters were at admission, except for CRP, which is the maximal value during the hospitalization. Abbreviations: MIS-C: multisystem inflammatory syndrome in children.

Appendix 1. STROBE checklist.

|  | Item No | Recommendation | Reported on page |
| --- | --- | --- | --- |
| **Title and abstract** | 1 | (*a*) Indicate the study’s design with a commonly used term in the title or the abstract | Page 1 |
|  |  | (*b*) Provide in the abstract an informative and balanced summary of what was done and what was found | Page 5 |
| Introduction | | |  |
| Background/rationale | 2 | Explain the scientific background and rationale for the investigation being reported | Page 6 |
| Objectives | 3 | State specific objectives, including any prespecified hypotheses | Page 7 |
| Methods | | |  |
| Study design | 4 | Present key elements of study design early in the paper | Page 8 |
| Setting | 5 | Describe the setting, locations, and relevant dates, including periods of recruitment, exposure, follow-up, and data collection | Page 8 |
| Participants | 6 | (*a*) Give the eligibility criteria, and the sources and methods of selection of participants. Describe methods of follow-up | Page 8-9 |
|  |  | (*b*) For matched studies, give matching criteria and number of exposed and unexposed | NA |
| Variables | 7 | Clearly define all outcomes, exposures, predictors, potential confounders, and effect modifiers. Give diagnostic criteria, if applicable | Page10-11 |
| Data sources/ measurement | 8* | For each variable of interest, give sources of data and details of methods of assessment (measurement). Describe comparability of assessment methods if there is more than one group | Page 8, 11 and 12 |
| Bias | 9 | Describe any efforts to address potential sources of bias | Page 10-11 |
| Study size | 10 | Explain how the study size was arrived at | Page 8 |
| Quantitative variables | 11 | Explain how quantitative variables were handled in the analyses. If applicable, describe which groupings were chosen and why | Page 12 |
| Statistical methods | 12 | (*a*) Describe all statistical methods, including those used to control for confounding | Page 12 |
|  |  | (*b*) Describe any methods used to examine subgroups and interactions | NA |
|  |  | (*c*) Explain how missing data were addressed | NA |
|  |  | (*d*) If applicable, explain how loss to follow-up was addressed | NA |
|  |  | (*e*) Describe any sensitivity analyses | NA |
| Results | | |  |
| Participants | 13* | (a) Report numbers of individuals at each stage of study—eg numbers potentially eligible, examined for eligibility, confirmed eligible, included in the study, completing follow-up, and analysed | Page 14 |
|  |  | (b) Give reasons for non-participation at each stage | NA |
|  |  | (c) Consider use of a flow diagram | NA |
| Descriptive data | 14* | (a) Give characteristics of study participants (eg demographic, clinical, social) and information on exposures and potential confounders | Page 14-15 |
|  |  | (b) Indicate number of participants with missing data for each variable of interest | Table 1 |
|  |  | (c) Summarise follow-up time (eg, average and total amount) | NA |
| Outcome data | 15* | Report numbers of outcome events or summary measures over time | Tables1, 2 and 3 |
| Main results | 16 | (*a*) Give unadjusted estimates and, if applicable, confounder-adjusted estimates and their precision (eg, 95% confidence interval). Make clear which confounders were adjusted for and why they were included | Tables1, 2 and 3 |
|  |  | (*b*) Report category boundaries when continuous variables were categorized | Tables1, 2 and 3 |
|  |  | (*c*) If relevant, consider translating estimates of relative risk into absolute risk for a meaningful time period | NA |
| Other analyses | 17 | Report other analyses done—eg analyses of subgroups and interactions, and sensitivity analyses | NA |
| Discussion | | |  |
| Key results | 18 | Summarise key results with reference to study objectives | Page 17 |
| Limitations | 19 | Discuss limitations of the study, taking into account sources of potential bias or imprecision. Discuss both direction and magnitude of any potential bias | Page 19-20 |
| Interpretation | 20 | Give a cautious overall interpretation of results considering objectives, limitations, multiplicity of analyses, results from similar studies, and other relevant evidence | Page 20 |
| Generalisability | 21 | Discuss the generalisability (external validity) of the study results | Page 17 |
| Other information | | |  |
| Funding | 22 | Give the source of funding and the role of the funders for the present study and, if applicable, for the original study on which the present article is based | Page 22 and Abstract |

*Give information separately for exposed and unexposed groups.

**Note:** An Explanation and Elaboration article discusses each checklist item and gives methodological background and published examples of transparent reporting. The STROBE checklist is best used in conjunction with this article (freely available on the Web sites of PLoS Medicine at http://www.plosmedicine.org/, Annals of Internal Medicine at http://www.annals.org/, and Epidemiology at http://www.epidem.com/). Information on the STROBE Initiative is available at http://www.strobe-statement.org.

Appendix 2. Members of the French Covid-19 Paediatric Inflammation Consortium and the French Pharmacovigilance network.

In addition to the authors, the following collaborators participated to the “French Covid-19 Pediatric Inflammation Consortium”

Maelle Selegny (Amiens); Lucas Jeusset, Aurelie Donzeau, Sophie Lety, Bertrand Leboucher (Angers); Agnes Baur (Annecy); Cristian Fedorczuk (Arcachon); Marion Lajus, Philippe Bensaid (Argenteuil); Yacine Laoudi (Aulnay Sous Bois); Charlotte Pons (Avignon); Anne-Cécile Robert, Camille Beaucourt (Besançon); Loïc De Pontual (Bondy); Muriel Richard, Etienne Goisque, Xavier Iriart, Olivier Brissaud, Pierre Segretin, Julie Molimard (Bordeaux); Marie-Clothilde Orecel, Gregoire Benoit (Boulognes Billancourt); Lucille Bongiovanni (Brest); Guerder Margaux, Robin Pouyau, Jean-Marie De Guillebon De Resnes, Ellia Mezgueldi, Fleur Cour-Andlauer, Come Horvat, Pierre Poinsot, Cecile Frachette, Antoine Ouziel, Yves Gillet (Bron); Catherine Barrey (Bry Sur Marne); Jacques Brouard, Florence Villedieu (Caen); Vathanaksambath Ro, Narcisse Elanga (Cayenne); Vincent Gajdos (Clamart); Romain Basmaci (Colombes); Hadile Mutar (Contamine sur Arve); Sébastien Rouget (Corbeil Essone); Elodie Nattes, Isabelle Hau, Sandra Biscardi, El Jurdi Houmam, Camille Jung (Créteil); Denis Semama, Frederic Huet (Dijon); Anne-Marie Zoccarato (Gap); Mayssa Sarakbi (Gonnesse); Guillaume Mortamet, Cécile Bost-Bru (Grenoble); Joachim Bassil (Laval); Caroline Vinit, Véronique Hentgen (Le Chesnay); Pascal Leroux, Valérie Bertrand, Caroline Parrod (Le Havre); Irina Craiu, Isabelle Kone-Paut, Philippe Durand, Pierre Tissiere, Caroline Claude, Guillaume Morelle, Tamazoust Guiddir, Charlotte Borocco (Le Kremlin-Bicêtre); Frédérique Delion (Les Abymes); Camille Guillot, Stéphane Leteurtre, François Dubos, Mylene Jouancastay, Alain Martinot, Valentine Voeusler (Lilles); Jane Languepin (Limoges); Nathalie Garrec, Arnaud Chalvon Demersay (Marne La Vallée); Aurélie Morand, Emmanuelle Bosdure, Noémie Vanel, Fabrice Ughetto, Fabrice Michel (Marseille); Caujolle Marie, Renaud Blonde, Jacqueline Nguyen (Mayotte); Olivier Vignaud, Caroline Masserot-Lureau, François Gouraud, Carine Araujo (Meaux); Tara Ingrao (Metz); Sanaa Naji (Mont de Marsans); Mohammed Sehaba (Montargis); Christine Roche (Montbrison); Aurelia Carbasse, Christophe Milesi (Montpellier); Mustapha Mazeghrane (Montreuil); Sandrine Haupt (Mulhouse); Cyril Schweitzer (Nancy); Benedicte Romefort, Elise Launay, Christèle Gras-Le Guen (Nantes); Ahmed Ali, Nathalie Blot (Neuilly Sur Seine); Antoine Tran, Anne Rancurel, Mickael Afanetti (Nice); Sophie Odorico (Nîmes); Deborah Talmud (Orléans); Anais Chosidow, Anne-Sophie Romain, Emmanuel Grimprel Marie Pouletty, Jean Gaschignard, Olivier Corseri, Albert Faye, Jean Gaschignard, Isabelle Melki, Camille Ducrocq, Cherine Benzoïd, Johanna Lokmer, Stéphane Dauger, Maryline Chomton, Anna Deho, Fleur Lebourgeois, Sylvain Renolleau, Fabrice Lesage, Florence Moulin, Laurent Dupic, Yael Pinhas, Agathe Debray, Martin Chalumeau, Véronique Abadie, Pierre Frange, Jeremie F Cohen, Slimane Allali, William Curtis, Zahra Belhadjer, Johanne Auriau, Mathilde Méot, Lucile Houyel, Damien Bonnet, Christophe Delacourt, Brigitte Bader Meunier, Pierre Quartier, Youssef Shaim, Laurence Baril, Samuel Crommelynck, Baptiste Jacquot (Paris); Philippe Blanc (Poissy); Natacha Maledon (Poitiers); Blandine Robert (Pontoise); Camille Loeile (Quimper); Clémence Cazau, Gauthier Loron (Reims); Simona Gaga (Remiremont); Cécile Vittot, Loubna El Nabhani (Rouen); François Buisson (Saumur); Muriel Prudent (Sens); Hugues Flodrops (St Denis, La Réunion); Fadhila Mokraoui, Simon Escoda (St Denis); Nina Deschamps (St Malot); Laurent Bonnemains, Sarah-Louisa Mahi, Clara Mertes, Joelle Terzic, Julie Helms (Strasbourg); Charlotte Idier (Tours); Soraya Chenichene, Nicoleta Magdolena Ursulescu (Trévenans); Gladys Beaujour (Villeneuve Saint Georges).

In addition to the authors, the following collaborators participated to the “French Pharmacovigilance network”:

Layal El Aridi (Brest), Hélène Géniaux (Limoge), Anthony Facile (Lyon), Tessa Pietri (Marseille), Pascale Palassin (Montpellier), Sylvine Pinel, Laurent Chouchana, Delphine Callot (Paris), Charlène Boulay (Rouen).
